# Monkey pox: A public health emergency

**DOI:** 10.1016/j.amsu.2022.104622

**Published:** 2022-09-12

**Authors:** Ahmed Kamal Siddiqi, Umeed Khan, Muhammad Adil

**Affiliations:** a Department of Medicine, Ziauddin Medical University, Karachi, Pakistan; b Department of Medicine, Jinnah Sindh Medical University, Karachi, Pakistan

Human monkeypox virus, a double-stranded DNA virus belongs to the Orthopoxvirus genus of the Poxviridae family with two genetic clades identified as West African and Central African [1]. First detected in 1958 when there was an outbreak of a vesicular disease among monkeys held in captivity being transported from Africa to Copenhagen, Denmark for research. The first case of monkeypox in humans was seen in a 9-year-old child belonging to the rural area of Zaire in August 1970, having smallpox-like vesicular lesions on the skin [1]. There have been reports of imported sporadic cases in Singapore, the UK, and the US from 2018 until recently in 2022, with the ongoing COVID-19 pandemic [2], an outbreak of monkeypox cases in the UK, other European and non-European countries was reported with confirmed cases of 2677 up to June 21, 2022 [3].

It is still not understood how the virus is transmitted to humans. It is assumed that the primary source of infection transmission is from animals to humans by handling monkeypox-infected animals. Secondary human-human transmission occurs through large respiratory droplets, contact with bodily fluids, lesions, and polluted surfaces such as clothing or linens [1,4]. Currently, many cases in the ongoing outbreak have been traced to sexual transmission, particularly among men who identify as gay or bisexual [5]. Initial symptoms begin with fever, headache, fatigue, and lymphadenopathy. Mucosal lesions in the mouth followed by lesions of skin on the face and extremities develop after 1–2 days. The lesions may vary in number from a small amount to thousands and may or may not spread to other parts of the body [6]. However, monkeypox cases reported in the current ongoing outbreak are atypical in nature with the characteristic rash developing in the genital and perianal areas with or without spreading to other body parts [7].

Even though, there is no specific treatment for monkeypox, the smallpox antivirals such as cidofovir, brincidofovir, and tecovirimat show activity against monkeypox with the latter two drugs having US FDA approval for use in treating smallpox. Most likely these medications would be reserved for treating severe cases or immunocompromised individuals and can be obtained through a public health department or the CDC [8]. The smallpox vaccines are believed to be effective in preventing monkeypox and as postexposure prophylaxis. JYNNEOS, which is a new generation smallpox vaccine, has an FDA indication for preventing monkeypox, and the older generation ACAM2000 used off-label can be used as well. Immediate post-exposure administration of the prophylactic vaccine can avoid infection or significantly reduce it. Alternative postexposure prophylaxis is vaccinia immune globulin in cases where the smallpox vaccine is contraindicated [8]. JYNNEOS vaccine can be administered in two doses, 28 days apart [9], and ACAM2000, which is a replication-competent live vaccinia virus vaccine can be administered as a single dose [10].

In conclusion, with the ongoing COVID-19 pandemic, monkeypox poses a great threat and has the potential to become a pandemic but with proper preventive strategies, the chances of containment of this disease increase to a great extent. Monkeypox, like most other viral infections, has symptomatic treatment. Educating patients and healthcare workers in areas where this disease is endemic is of utmost importance. People with knowledge about monkeypox can practice social distancing and other preventive strategies such as wearing a mask, washing hands regularly, and practicing safe sex. Healthcare workers with adequate knowledge about this disease can devise treatment plans and diagnostic strategies to help curb this virus among the masses. The infected individual must remain in isolation, wear a surgical mask, and keep the lesions covered as much as reasonably possible until all lesion crusts have naturally fallen off and a new skin layer has formed. Hospitals in endemic areas must acquire vaccines and medicines in time and make them available to the o masses. Government should encourage people to get vaccinated and educate and aware the general population about this through numerous social media platforms including TV, social media platforms, and messages on mobile phones. In areas where access to these things is lesser, mass campaigns need to be organized and people need to be taught in simple language, easy to understand.

## Ethical approval

N/A.

## Sources of funding

I declare no financial or competing interests.

## Author contributions

Ahmed Kamal Siddiqi: Study concept and design.

Umeed Khan: Acquisition of the data and drafting of the manuscript.

Muhammad Adil Obaid: Acquisition of the data and drafting of the manuscript.

## Conflicts of interest

I declare no financial or competing interests.

## Registration of research studies

1. Name of the registry: N/A/
2. Unique Identifying number or registration ID: N/A.
3. Hyperlink to your specific registration (must be publicly accessible and will be checked): N/A.

## Guarantor

Ahmed Kamal Siddiqi Ahmedsiddiqi2020@gmail.com +92–03121222808.

## Consent

N/A.
